# Identification of a Novel Partitivirus of *Trichoderma harzianum* NFCF319 and Evidence for the Related Antifungal Activity

**DOI:** 10.3389/fpls.2018.01699

**Published:** 2018-11-20

**Authors:** Jeesun Chun, Han-Eul Yang, Dae-Hyuk Kim

**Affiliations:** ^1^Institute for Molecular Biology and Genetics, Chonbuk National University, Jeonju, South Korea; ^2^Department of Bioactive Material Sciences, Chonbuk National University, Jeonju, South Korea

**Keywords:** *Trichoderma harzianum*, mycovirus, partitivirus, mycoparasitism, antifungal activity

## Abstract

We have reported 15 agarose gel band patterns of double-stranded RNA (dsRNA) from *Trichoderma* spp. We describe herein that band pattern IX in *Trichoderma harzianum* NFCF319, which appeared to be a single band but consisted of two dsRNAs of similar size, was identified as a novel mycovirus, designated *Trichoderma harzianum* partitivirus 1 (ThPV1). The larger segment (dsRNA1) of the ThPV1 genome comprised 2,289 bp and contained a single open reading frame (ORF) encoding an RNA-dependent RNA polymerase (RdRp). The smaller segment (dsRNA2) consisted of 2,245 bp with a single ORF encoding a capsid protein (CP). Evaluation of the deduced amino acid sequence and phylogenetic analysis indicated that ThPV1 is a new member of the genus *Betapartitivirus* in the family *Partitiviridae*. Curing of virus infection by single-sporing generated 31 virus-free single-spore clones. No significant differences in growth rate, conidia production, or pigmentation were observed between ThPV1-infected and -cured isogenic strains. In addition, comparison of the newly ThPV1-transmitted isolates with their ThPV1-cured parental strain showed no significant difference in colony morphology or pigmentation. However, inhibition of growth in co-cultured *Pleurotus ostreatus* and *Rhizoctonia solani* by *T. harzianum* was increased in ThPV1-containing strains compared with ThPV1-cured isogenic strains. Moreover, β-1,3-glucanase activity was significantly increased in the ThPV1-containing strains. However, no difference in chitinase activity was observed, suggesting that ThPV1 regulates the activity of a specific fungal enzyme.

## Introduction

Mycoviruses, i.e., fungal viruses, are widespread and have been found in all major taxa of filamentous fungi and yeasts (Van Alfen, 1986; Nuss and Koltin, 1990; Wickner, 1992). The majority of characterized mycoviruses have a double-stranded RNA (dsRNA) genome, although others have single-stranded RNA (ssRNA) and DNA genomes. Mycoviruses of seven families, *Chrysoviridae, Endornaviridae, Megabirnaviridae, Quadriviridae, Partitiviridae, Reoviridae*, and *Totiviridae* have dsRNA genomes, while those of six families, *Alphaflexiviridae, Barnaviridae, Gammaflexiviridae, Hypoviridae, Narnaviridae*, and *Mymonaviridae* have ssRNA genomes according to a report by the International Committee on the Taxonomy of Viruses (ICTV) in 2016 (Ghabrial et al., 2015). Using multi-omics techniques, a large number of novel mycoviruses are expected to be discovered and characterized from various fungi. Recently, we reported the presence of a variety of dsRNA elements in many strains of *Trichoderma* spp. causing a green mold disease in shiitake mushroom (*Lentinula edodes*) and confirmed the presence of novel mycoviruses in these *Trichoderma* spp. (Yun et al., 2016). Although infection of fungi by many mycoviruses is asymptomatic or cryptic (Ghabrial, 1998), there are many cases of mycoviruses inducing viral-specific symptoms in the host and reduced fungal virulence, known as hypovirulence, is one of representative examples in phytopathogenic fungi (Nuss, 2005).

Members of the genus *Trichoderma* are fast-growing ubiquitous soil fungi. The evident success of *Trichoderma* species is due to the characteristic mechanisms of *Trichoderma* to survive and proliferate, which include an aggressive ability to inhibit other fungi and production of various enzymes for the degradation of complex carbohydrates (Samuels and Hebbar, 2015). These activities have been exploited for human benefit; indeed, some *Trichoderma* species are used as biocontrol agents, and in the production of commercial enzymes and secondary metabolites, and are endosymbionts for their host plant (Kubicek and Penttila, 1998; Sivasithamparam and Ghisalberti, 1998; Hatvani et al., 2002; Howell, 2003). *T. harzianum* is probably the most commonly cited species and is widely recognized as a potential biocontrol component in controlling common soil-borne plant pathogens (i.e., *Fusarium, Pythium*, and *Rhizoctonia*), but is also reported as the causal agent of green mold disease in cultivated mushrooms (Papavizas, 1985; Tokimoto, 1985; Ulhoa and Peberdy, 1992; Seaby, 1998; Kredics et al., 2010). Thus, changes in the beneficial or pathological characteristics of *T. harzianum* impact the industrial application or prevention of this fungus. We have reported the presence of dsRNA in *T. harzianum*; however, the nature and biological function of the dsRNA are unclear.

In this study, we characterized the dsRNA of a novel mycovirus and assessed its biological effects on *T. harzianum*.

## Materials and Methods

### Fungal Strains and Growth Condition

Fungal strains were maintained at 25°C in the dark on PDA. Virus-containing and -cured *T. harzianum* strains were maintained by the hyphal-tipping technique.

### Nucleic Acid Extraction and Viral Genome Sequencing

dsRNA extraction and Northern hybridization analysis were performed as described by Park et al. (2008). Purified dsRNA was subjected to cDNA library construction and genome sequencing on the Illumina Hiseq 2000 platform (Macrogen Inc., Seoul, South Korea). The Illumina adapter sequence reads were quality checked by FastQC and trimmed by Trimmomatic (ver. 0.32). Qualified reads were assembled to generate contigs by Trinity and abundance was estimated using RSEM software to calculate the FPKM-values.

### Rapid Amplification of cDNA Ends (RACE) Analysis

RNA ligase-mediated rapid amplification of cDNA ends (RLM-RACE) was performed to determine the 5′- and 3′-terminal sequences of dsRNA using an RLM-RACE kit (Ambion, Austin, TX, United States). Purified dsRNA was denatured in dimethylsulfoxide and treated with calf intestine alkaline phosphatase (CIP) and tobacco acid pyrophosphatase (TAP) to remove free 5′ phosphates and cap structures. The 5′ RNA adapter oligonucleotide (5′-GCUGAUGGCGAUGAAUGAACACUGCGUUUGCUGGCUU UGAUGAAA-3′) was ligated to the decapped RNA using T4 RNA ligase. The ligates were subjected to random-primed reverse transcription and the 5′ end of a specific sequence was amplified. The 3′ terminus of dsRNA was ligated to a 3′ RACE adapter oligonucleotide (5′-GCGAGCACAGAATTAATACGACTCACTATAGGT12VN-3′) and subjected to RT-PCR. The resulting cDNA was amplified by PCR to determine the 3′ end sequence.

### Sequence Analysis

Phylogenetic trees were constructed by the maximum-likelihood methods (Fitch, 1971) with the software package MEGA7 (Kumar et al., 2016) after performing multiple sequence alignments using CLUSTAL X (ver. 2.1) (Thompson et al., 1997).

### Purification of Virus Particles and Transmission Electron Microscopy

*Trichoderma harzianum* betapartitivirus 1 (ThPV1) particles were purified by the method of Wang et al. (2014) and subjected to sucrose gradient ultracentrifugation. The structure of the virus-like particles was visualized by TEM on an H-7650 instrument installed in the Center for University-Wide Research Facilities (CURF) at Chonbuk National University (Hitachi, Tokyo, Japan) after negative staining with 2% uranyl acetate.

### Antifungal Activity Assay

Conidial suspensions (1 × 10^5^ spores/ml) of *T. harzianum* NFCF319 were inoculated in 150 ml of potato dextrose broth (PDB) and incubated in shaking flasks for 2 days at 25°C and 180 rpm. The culture medium was passed through a 0.2 μm membrane filter and tested for each filtrate diluted with PDB containing 10^-1^, 10^-2^, and 10^-3^ of the original filtrate. *Pleurotus ostreatus* (ASI No. 2792) and *Rhizoctonia solani* AG-1 (KACC40101) were cultured by placing mycelial plugs in the center of PDA containing each filtrate, and colonial growth was assessed as the mean radial area.

### Chitinase and β-1,3-Glucanse Assay

Culture supernatants were harvested and subjected to chitinase assays according to the manufacturer’s instructions (Sigma, St. Louis, MO, United States). β-1,3-glucanase assays were performed in 0.05 M sodium citrate buffer (pH 4.5) with β-1,3-glucan for 2 h. The reaction was stopped by heating at 100°C for 5 min, and the amount of reducing sugar liberated was measured using neocuproine. One unit (U) of β-1,3-glucanase activity was defined as the amount of an enzyme that produced 1 μmol of reducing sugar per minute under the assay conditions.

### Transformation

Protoplasts were prepared as described previously (Penttilä et al., 1987). Transformation using protoplasts was performed as described previously, with slight modification (Churchill et al., 1990; Kim et al., 2002). Briefly, polyethylene glycol (PEG) 6,000 (Sigma) was used instead of PEG 4,000 when transforming DNA was mixed with protoplasts. The protoplasts were transformed with pDH25, which carried the hygromycin phosphotransferase gene cassette (*hph*) (Cullen et al., 1987). Transformants were selected from agar plates that were supplemented with 150 μg/ml hygromycin B (Calbiochem; Merck, Darmstadt, Germany), passaged three or four times on selective medium, and single-spore isolated as described previously (Kim et al., 2002). PCR and Southern blot analysis were conducted with genomic DNA from the transformants to confirm the hygromycin B resistance gene.

### Transmission of dsRNA Virus

Virus transmission was performed as described previously (Kim et al., 2013). Briefly, mycelial plugs of the virus-containing strain were placed on PDA medium adjacent to mycelial plugs of the virus-free hygromycin-B-resistant recipient. After 3 days of co-culture, putatively fused mycelia at the recipient border between each pair of strains were transferred to hygromycin-containing PDA, successively transferred to fresh hygromycin-containing medium at least three times, and examined for the presence of virus. Then, the virus-containing mycelial progenies were single-spored to select for the virus-infected recipient transformants. The presence of mycovirus was confirmed by purification of dsRNA from the single-spored isolates.

## Results

### Sequence Determination and Genome Organization

**FIGURE 1:**
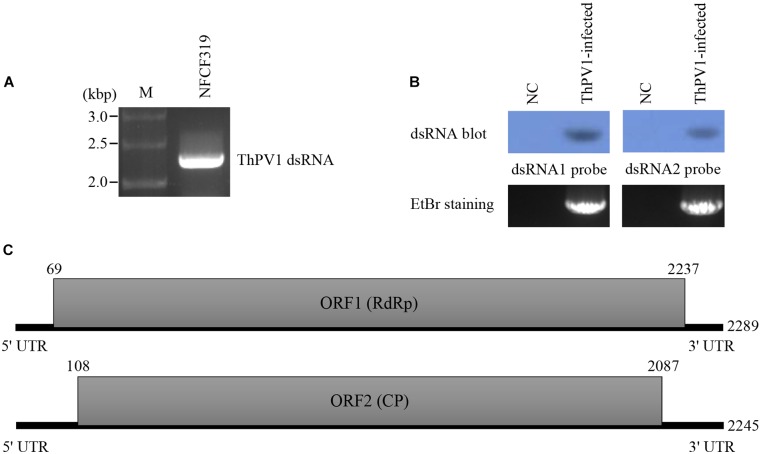
Structure of the *T. harzianum* partitivirus 1 (ThPV1) genomic double-stranded RNAs (dsRNAs). **(A)** Agarose gel electrophoresis of ThPV1 dsRNA. Genomic dsRNA was extracted from virus-infected *T. harzianum* NFCF319. Lane M, DNA size standard. **(B)** Northern blot analysis of ThPV1 dsRNA1 and dsRNA2. RNAs were hybridized with probes for dsRNA1 and dsRNA2. NC: negative control (ThPV1-free). **(C)** Schematic representation of ThPV1 dsRNA segments. Shaded boxes, open reading frames (ORFs) encoding RNA-dependent RNA polymerase (RdRp) and capsid protein (CP). Numbers indicate the total lengths of the ThPV1 genome segments and the positions of the start and stop codons.

The dsRNA extracted from the mycelia of *T. harzianum* NFCF319 was resolved by 1% agarose gel electrophoresis, and distinctive bands of approximately 2.3 kb were excised (Figure 1A). The gel-purified dsRNAs were subjected to construct an RNA-Seq library followed by sequencing using Illumina HiSeq 2000. A total of 109 assembled reads with significant fragments per kilobase of transcript per million mapped reads (FPKM) values were obtained. A homology search of the assembled reads suggested the presence of contigs with high similarity to the known viral sequences of RNA-dependent RNA polymerase (RdRp) and capsid protein (CP). Based on the sequence analysis, RT-PCR using the corresponding primer pairs (see Supplementary Table S1) based on two representative contigs (i.e., one for RdRp and the other for CP) resulted in PCR amplicons of the expected sizes, which were subjected to sequence for verification. Northern blot analysis using the dsRNA extracts and probes (see Supplementary Table S1) constructed from PCR amplicons of portions of RdRp and CP revealed a specific hybridization band indicating that the excised dsRNA bands were double bands with similar sizes corresponding each of RdRp and CP. Thus, the analysis of the dsRNA sequences and Northern hybridization indicated that the mycoviral genome is divided into two dsRNA segments (dsRNA1 and dsRNA2) of similar size (Figure 1B). Rapid amplification of cDNA ends (RACE) was conducted to determine the full-length of the dsRNAs.

The complete genome sequences indicated that the dsRNA1 segment was of 2,289 bp with a GC content of 45.1%, and dsRNA2 was of 2,245 bp with a GC content of 49.5%. These sequences were deposited in GenBank (accession number MG973751 and MG973752, respectively). Each segment contains a long open reading frame (ORF) and ORF1 and 2 were on the coding strand of dsRNA1 and dsRNA2, respectively. RACE analysis indicated that the 5′ untranslated region (UTR) of the coding strand of dsRNA1 was 68 nt long and the 3′ UTR was 52 nt long. In addition, the coding strand of dsRNA2 contained a 107-nt-long 5′ UTR and 158-nt-long 3′ UTR. The structure of genome organization of dsRNA1 and dsRNA2 are displayed in Figure 1C.

The sequence of the 5′-termini of both dsRNA1 and 2 (GAACAAGG) were similar to the known consensus sequence of the betapartitivirus GAWWUWNU (N, any nt; W, A or U) and well-matched those of Heterobasidion partitivirus 2 (HetPV2) (Vainio et al., 2011). In addition, characteristic A-rich regions in the 3′-termini were preserved in both dsRNA1 [31 (A) residue in the 3′-terminal 50 nt] and dsRNA2 [48 (A) residue in the 3′-terminal 50 nt]. Like other fungal partitiviruses showing the characteristics of lengths of 5′- and 3′-non-translated region (NTR), longer 3′-NTR than 5′-NTR was found in dsRNA2 and longer 3′-NTR in dsRNA2 than dsRNA1 was found. Therefore, these results indicated that the obtained sequences were of the full-length of dsRNA1 and 2.

**FIGURE 2:**
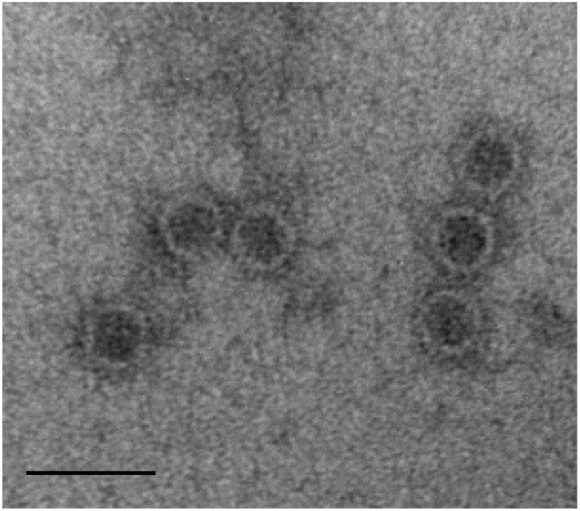
Morphology of ThPV1 particles. Purified virus particles were negative-stained with 2% uranyl acetate and examined by transmission electron microscopy (TEM). Scale bar, 50 nm.

Viral particles were visualized by transmission electron microscopy (TEM). The particles were spherical and of diameter 28–30 nm (Figure 2).

### Phylogenic Analysis of ORF1 and ORF2

**FIGURE 3:**
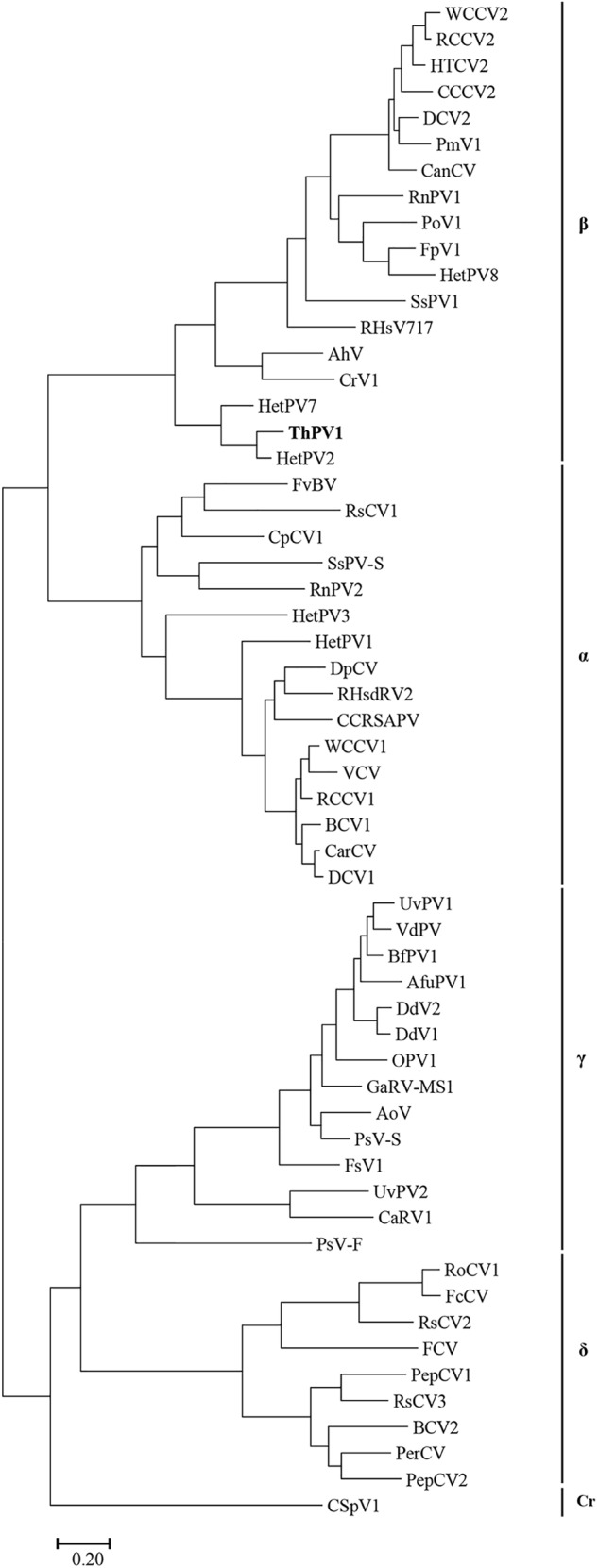
Phylogenetic analysis of ThPV1. Maximum-likelihood (ML) tree based on the RdRp amino acid sequences of *Partitiviridae*. α, *Alphapartitivirus*; β, *Betapartitivirus*; γ, *Gammapartitivirus*; δ, *Deltapartitivirus*; Cr, *Cryspovirus*. See Supplementary Table S2 for the partitivirus names and their GenBank numbers. Scale bar, number of substitutions per nucleotide site.

The deduced amino acid sequence of ORF1 consisted of 722 amino acids with a predicted molecular mass of 79.4 kDa. Homology search of the deduced amino acid sequence revealed high similarity to the known sequences of RdRp of Heterobasidion partitivirus 2 (HetPV2) [GenBank accession number, HM565953; E-value, 0; AA identity, 589/722 (81%)]. Phylogenetic analysis using the top ranked similar sequences indicated that our RdRp clustered with the genus *Betapartitivirus*, and this was supported by significant bootstrap values. We next used the above sequence information for taxonomic reorganization of the family *Partitiviridae* (Vainio et al., 2015). The extensive analysis showed that our RdRp clustered with HetPV2 and Heterobasidion partitivirus 7 (HetPV7) (Figure 3).

ORF2 was deduced to encode a 659-amino acid protein of predicted molecular mass 72.5 kDa. Homology search showed high similarity to the CP of HetPV2 [GenBank accession number, HM565954; E-value 2e - 65; AA identity, 444/655 (68%)]. Phylogenetic analysis of CP indicated that our sequence clustered strongly to those of HetPV2 and HetPV7. Sequence similarity, genome organization, and phylogenetic analysis indicated that the dsRNAs are genome segments of a new member of the genus *Betapartitivirus* in the family *Partitiviridae*. Thus, we named our dsRNA as *Trichoderma harzianum* betapartitivirus 1 (ThPV1). Based on the ICTV species demarcation criteria for the genus *Betapartitivirus* in the family *Partitiviridae* (≤90% and ≤80% amino acid identities in the RdRp and CP, respectively), we conclude that ThPV1 represents a novel species.

### Characteristics of Mycovirus-Cured and -Containing Strains

Virus-cured isogenic strains were obtained by producing single-spored progenies followed by dsRNA purification. Spores were harvested from 7-day-old culture plates containing actively growing *T. harzianum* NFCF319, and 50 expected colony-forming units (CFUs) were spread on fresh potato dextrose agar (PDA) plate. Of 39 single-spored progenies, ThPV1 dsRNA bands were missing in 31. RT-PCR using gene specific primer pairs did not amplify any band and no hybridization band was observed either, which indicated that ThPV1 was cured in those selected strains. Virus-cured strains were successively sub-cultured weekly and subjected to dsRNA extraction every 4 weeks for 6 months. None of the cured-strains harbored ThPV1 dsRNA, suggesting that they were stable. To increase the biological replicates, 3 out of 31 cured strains (*T. harzianum* NFCF319-Vf1, -Vf2, and -Vf3), and 3 from 7 vertically transmitted virus-containing clones (*T. harzianum* NFCF319-V1, -V2, and -V3) were randomly selected for further analysis.

**FIGURE 4:**
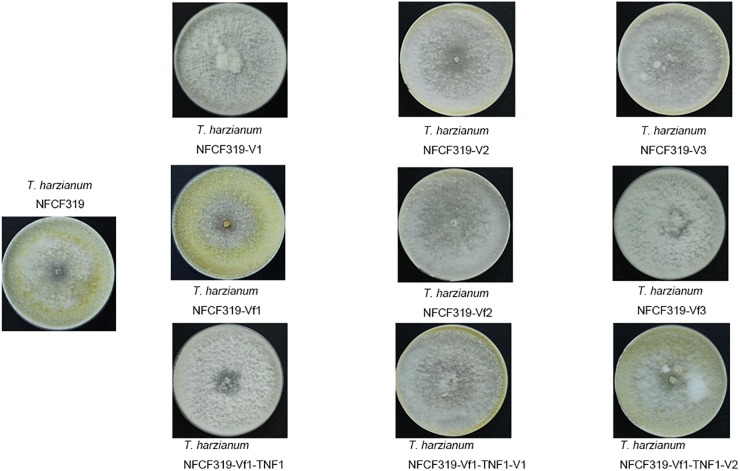
Colony morphology of the original ThPV1-infected (*T. harzianum* NFCF319), three single-spored ThPV1-containing (*T. harzianum* NFCF319-V1, -V2, and -V3), isogenic ThPV1-cured (*T. harzianum* NFCF319-Vf1, -Vf2, and -Vf3), ThPV1-free hygromycin B resistant transformant (*T. harzianum* NFCF319-Vf1-TNF1), and isogenic ThPV1-transmitted (*T. harzianum* NFCF319-Vf1-TNF1-V1 and -V2) isolates.

No difference in growth rate was observed among the four ThPV1-infected *T. harzianum* isolates, i.e., between the original ThPV1-infected *T. harzianum* NFCF319 strain and its three single-spored virus-containing progenies *T. harzianum* NFCF319-V1, -V2, and -V3. In addition, no significant differences in growth rate or colony morphology were detected between the ThPV1-infected and -cured strains. Although the time of appearance and intensity of the pigmentation varied among individual cultures, a characteristic diffusing yellow pigment developed in all strains during cultivation. In addition, all strains produced similar numbers of gray–green conidia per plate, dispersed in concentric rings (Figure 4).

### Biological Comparison of Mycovirus-Cured and -Containing Strains

**FIGURE 5:**
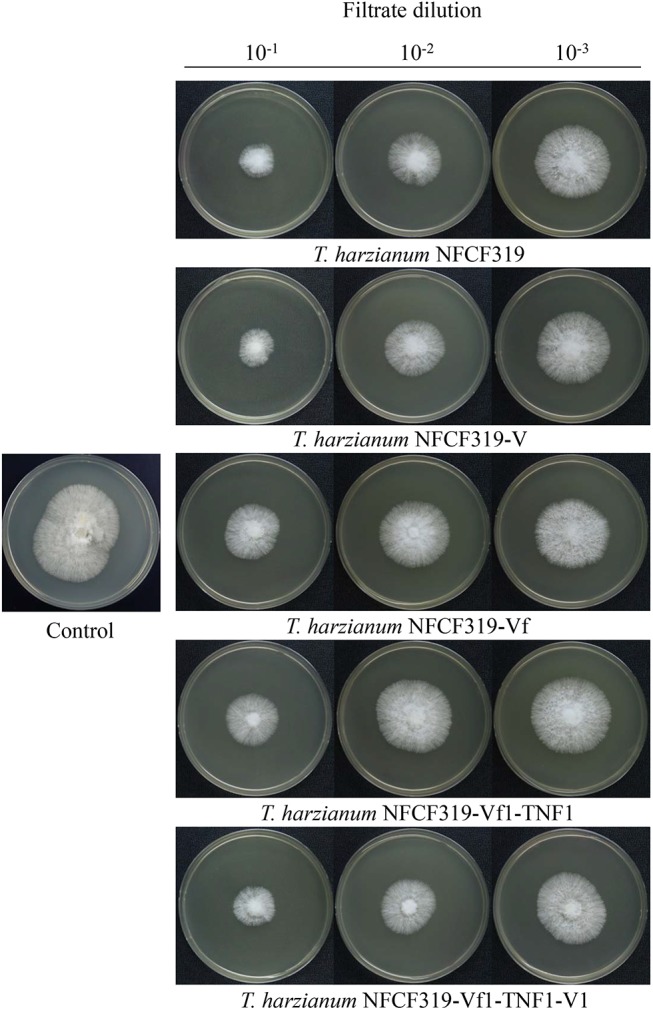
Colony morphology of *P. ostreatus* supplemented with culture filtrates of ThPV1-containing and -free strains. Inhibition of the growth of colonies of *P. ostreatus* with culture filtrate supplementation was compared to without supplementation (control). Numbers at top indicate the dilution factors of the culture filtrates.

**FIGURE 6:**
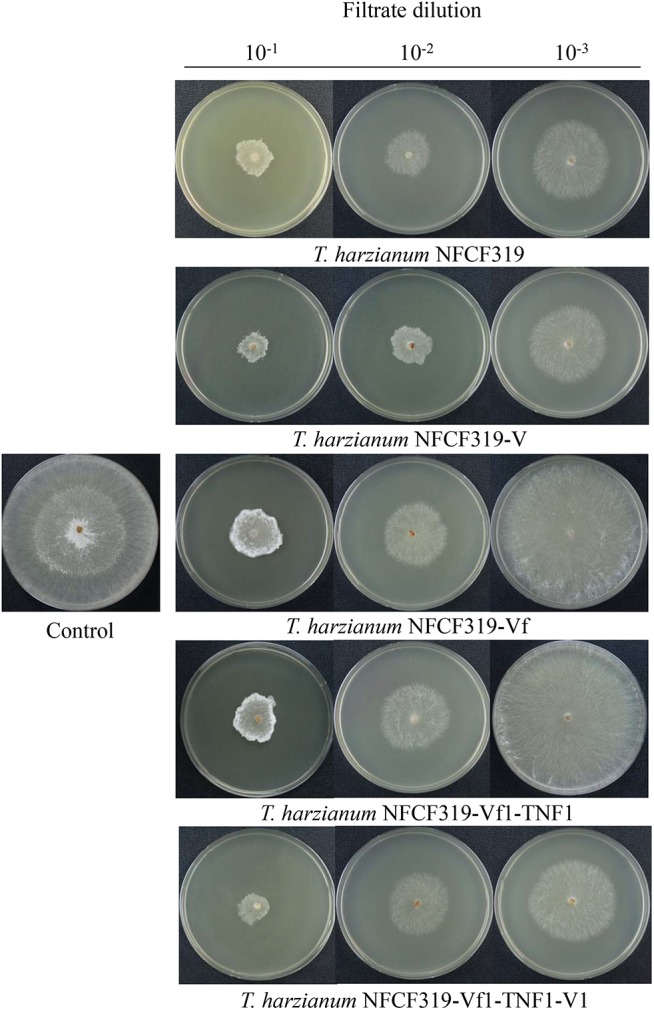
Colony morphology of *R. solani* supplemented with culture filtrates of ThPV1-containing and -free strains. Inhibition of the growth of colonies of *R. solani* with culture filtrate supplementation was compared to without supplementation (control). Numbers at top indicate the dilution factors of the culture filtrates.

The antagonistic activities of the virus-cured strains against the oyster mushroom, *P. ostreatus*, were measured using culture filtrates of the ThPV1-containg strains (*T. harzianum* NFCF319 and *T. harzianum* NFCF319-V1, -V2, and -V3) and ThPV1-cured strains (*T. harzianum* NFCF319-Vf1, -Vf2, and -Vf3). Significant differences in the growth rate of *P. ostreatus* were observed between medium without and with supplementation with the culture filtrates of *T. harzianum*, regardless of ThPV1 infection. Moreover, significant differences in growth rate were observed between medium supplemented with the culture filtrates of virus-cured and -containing strains. The growth rate was significantly reduced in medium supplemented with culture filtrate of the virus-containing strain (Figure 5 and Table 1). Colonial growth inhibition was also examined using the plant pathogenic fungus *R. solani* AG-1 (Figure 6 and Table 1). Significant differences in colony growth were observed in supplemented compared with non-supplemented medium. Furthermore, the culture filtrates of the virus-containing strains (*T. harzianum* NFCF319 and *T. harzianum* NFCF319-V1, -V2, and -V3) inhibited the growth of the oyster mushroom and a phytopathogenic fungus more strongly. To examine the effect of the heat-inactivated culture filtrate on fungal growth inhibition, colonial growth inhibition of *R. solani* AG-1 was tested using medium supplemented with the heat-inactivated culture filtrate. Colonial growth was inhibited by supplementation with the heat-inactivated culture filtrate, but the effect was not as intense as it was with the original culture filtrate. Interestingly, heat inactivation altered growth inhibition by the culture filtrates of the virus-containing strains to the level of the virus-cured strains.

**Table 1:** Growth inhibition due to the supplementation of culture filtrates.

Dilution rate	Strains	Growth inhibition of *P. ostreatus* (%)	Growth inhibition of *R. solani* (%)
1:10	ThPV1-infected	90.29^c^	94.78^c^
	ThPV1-containing	91.33^c^	95.49^c^
	Isogenic ThPV1-cured	78.82^b^	86.87^b^
	ThPV1-free hygromycin B resistant transformant	81.06^b^	86.85^b^
	Isogenic ThPV1-transmitted	91.04^c^	95.89^c^
1:100	ThPV1-infected	82.03^c^	87.01^c^
	ThPV1-containing	81.64^c^	87.55^c^
	Isogenic ThPV1-cured	66.92^b^	80.98^b^
	ThPV1-free hygromycin B resistant transformant	66.66^b^	80.53^b^
	Isogenic ThPV1-transmitted	81.56^c^	87.51^c^
1:1000	ThPV1-infected	66.39^b^	78.73^c^
	ThPV1-containing	67.06^b^	77.08^c^
	Isogenic ThPV1-cured	66.58^b^	5.23^b^
	ThPV1-free hygromycin B resistant transformant	65.58^b^	5.36^b^
	Isogenic ThPV1-transmitted	67.05^b^	76.61^c^


*ThPV1, *Trichoderma harzianum* partitivirus 1. Growth inhibition by metabolites from the original ThPV1-infected (*T. harzianum* NFCF319), three single-spored ThPV1-containing (*T. harzianum* NFCF319-V1, -V2, and -V3), isogenic ThPV1-cured (*T. harzianum* NFCF319-Vf1, -Vf2, and -Vf3), ThPV1-free hygromycin B resistant transformant (*T. harzianum* NFCF319-Vf1-TNF1), and isogenic ThPV1-transmitted (*T. harzianum* NFCF319-Vf1-TNF1-V1 and -V2) isolates. Numbers are the relative inhibition rates (%) compared to without fungal metabolite supplementation. Data were analyzed by ANOVA followed by *post hoc* Tukey’s with the level of significance at *p* < 0.05. The same letters at the top of the relative inhibition rate indicate no significant difference between isolates.*

**FIGURE 7:**
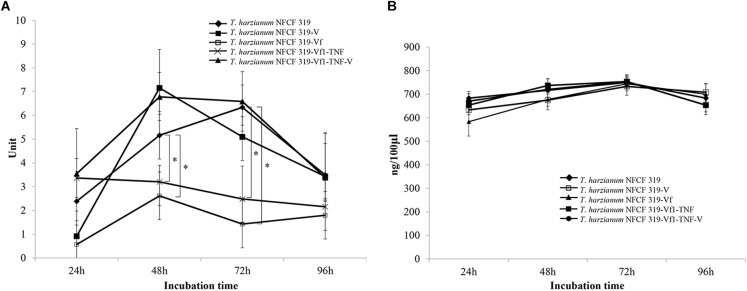
Enzyme activities in culture filtrates of ThPV1-containing and -free strains. The β-1,3-glucanase **(A)** and chitinase **(B)** activities of the original ThPV1-infected (*T. harzianum* NFCF319), mean of three single-spored ThPV1-containing (*T. harzianum* NFCF319-V1, -V2, and -V3), mean of isogenic ThPV1-cured (*T. harzianum* NFCF319-Vf1, -Vf2, and -Vf3) and ThPV1-free hygromycin B resistant transformant (*T. harzianum* NFCF319-Vf1-TNF1), and mean of two isogenic ThPV1-transmitted (*T. harzianum* NFCF319-Vf1-TNF1-V1 and -V2) isolates. The error bars indicate the standard deviations for three repeats per isolate. Student’s *t*-test (^∗^*P* < 0.05) was used to analyze the data between the *T. harzianum* NFCF319 and other strains.

The activities of two representative antifungal enzymes, β-1,3-glucanase and chitinase, were analyzed. The enzyme activity of the culture filtrate was monitored at 24 h intervals for 4 days. As shown in Figure 7, the time course of the β-1,3-glucanase activity was significantly reduced in the culture filtrate of the virus-cured strains of *T. harzianum* NFCF319 (*T. harzianum* NFCF319-Vf denotes the mean of three replicates of each of the NFCF319-Vf1, -Vf2, and -Vf3 isolates). Interestingly, there was no difference in chitinase activity between the virus-infected and -cured strains.

To validate the viral effect on the antifungal activity, we performed a “plus” experiment, i.e., we artificially transmitted ThPV1 into a virus-cured strain via anastomosis between virus-infected and -cured strains and analyzed the antifungal activity of the resulting virus-infected recipient strains. First, we transformed the cured strain of *T. harzianum* NFCF319-Vf1 using the hygromycin B resistance cassette to differentiate the ThPV1-transmitted progeny from the parental virus-containing *T. harzianum* NFCF319, because all cured strains were isogenic to their parental strain, i.e., the genetic background of all strains was identical, except for the presence or absence of mycovirus. Among 11 transformants showing stable resistance to hygromycin B after successive transfer to selective medium, the transformant *T. harzianum* NFCF319-Vf1-TNF1 was selected randomly and we observed no phenotypic changes from the parental *T. harzianum* NFCF319-Vf1 strain (Figure 4). After prolonged paired growth of the viral donor (*T. harzianum* NFCF319) and viral recipient (*T. harzianum* NFCF319-Vf1-TNF1) strains, several hyphae at the recipient border were collected and cultured independently on the selective medium, and the presence of ThPV1 was analyzed. Two independent single-spored virus-transmitted hyphal progenies were obtained. One of each single-spored progeny (*T. harzianum* NFCF319-Vf1-TNF1-V1 and -V2) containing ThPV1 was selected for further analysis (Figure 4). As shown in Figures 5, 6, medium supplemented with the culture filtrates of virus-transmitted *T. harzianum* NFCF319-Vf1-TNF1-V resulted in significant differences in the growth inhibition of *P. ostreatus* and *R. solani*. The level of inhibition was similar to that of the original virus-infected *T. harzianum* NFCF319 and significantly greater than those of virus-cured *T. harzianum* NFCF319-Vf and its transformant *T. harzianum* NFCF319-Vf1-TNF. In addition, significant increases in β-1,3-glucanase activity were observed in *T. harzianum* NFCF319-Vf1-TNF1-V compared with the parental virus-cured transformant *T. harzianum* NFCF319-Vf1-TNF (Figure 7). The β-1,3-glucanase activity in *T. harzianum* NFCF319-Vf1-TNF1-V was comparable to that of the original virus-infected *T. harzianum* NFCF319 (Figure 7). The enzyme activity profiles of β-1,3-glucanase were similar among the virus-containing strains. The enzyme activity of the virus-containing strains reached a peak at 48 or 72 h and then decreased gradually to 96 h. The enzyme activity profiles of β-1,3-glucanase were similar across the virus-cured strains. However, the levels of the enzyme activity of the virus-cured strains were significantly lower at 48 and 72 h of incubation and remained constant until 96 h. No difference in chitinase activity was observed among all strains tested.

## Discussion

The two-segmented dsRNA genome (each with one ORF) and isometric particles of 28–30 nm diameter suggest that ThPV1 belongs to the family *Partitiviridae*. According to the ICTV in 2016, the family *Partitiviridae* comprises the genera *Alphapartitivirus, Betapartitivirus, Gammapartitivirus, Deltapartitivirus*, and *Cryspovirus* (Nibert et al., 2014; Lefkowitz et al., 2017). The sizes of the segments and the molecular masses of the encoded proteins of ThPV1 are within the normal range of the genus *Betapartitivirus*, which are characteristically larger than those of other genera (Nibert et al., 2014). In addition, the high pairwise-identity scores of the amino acid sequences of encoded RdRp (81% to RdRp of HetPV2) and CP (68% to CP of HetPV2) to other known *Partitiviridae* (Nibert et al., 2014), and the clustering of ThPV1 with other members of the genus *Betapartitivirus*, indicates that ThPV1 is a new member of the genus *Betapartitivirus*.

Virus curing by single-spore isolation was successful; i.e., 79.5% of the resulting single-spore colonies were ThPV1-free. In addition, the ThPV1-free proportion was similar (20–84%) among three independent experiments. Therefore, the rate of vertical transmission of ThPV1 to mitotic progenies, conidia, of host fungus was low, although a considerable proportion of conidia showed vertical transmission of ThPV1. Like other mycoviruses, partitiviruses are generally considered to be transmitted vertically during cell division and horizontally during intimate cell-cell contact. Heterobasidion partitiviruses, which are closely related to ThPV1, could be transmitted vertically to basidiospores and conidiospores and horizontally via hyphal contact (Ihrmark et al., 2002, 2004). The mitotic stability measured by inheritance of mycoviruses during asexual conidiation differed depending on the virus–fungus interaction. The mitotic inheritance of ThPV1 during asexual conidiation was less stable than that of Cryphonectria hypovirus 1 (CHV1) in *C. parasitica* (Suzuki et al., 2003; Prospero et al., 2006), and of mycoreovirus 1 (MyRV1) in *C. parasitica* (Sun et al., 2006), but comparable to dsRNAs in the basidiomycetes *H. annosum* (Ihrmark et al., 2002). The low transmission rate of ThPV1 into conidia is exceptional considering that most vertical transmission of dsRNA into asexual spores of ascomycetes occurs at a markedly higher rate. Indeed, the recently characterized Trichoderma atroviride mycovirus 1 (TaMV1) has a very high transmission rate (33/38) (Lee et al., 2017). Therefore, the low transmission rate of ThPV1 into conidia is attributable to the intrinsic characteristics of the virus–fungus (ThPV1–*T. harzianum*) interaction.

Considering their persistent lifestyles and direct cell-to-cell transmission, partitiviruses has few, if any, deleterious effects on host cells (Nibert et al., 2014). However, others have reported negative or positive effects of partitiviruses on their host (Suzuki et al., 2001; Márquez et al., 2007; Jenkins et al., 2008; Kanematsu et al., 2010; Vainio et al., 2010, 2017; Hyder et al., 2013; Xiao et al., 2014; Zheng et al., 2014). ThPV1 did not influence the mycelial growth, colony morphology, pigmentation, or conidiation of its host fungus. However, ThPV1 significantly altered the activities of antifungal enzymes potentiating the biocontrol function of its host fungus *T. harzianum*. It is interesting to see that, regardless of virus-containing or -cured, supplementation of culture filtrates resulted in significantly increased antifungal activities compared to the non-supplementation; furthermore, the heat-treated culture filtrate of the ThPV1-infected strain showed significantly reduced antifungal activities similar to the level of ThPV1-free strain. These results clearly suggested that the antifungal activities were attributable to both heat-stable and -sensitive metabolites. Considering the facts that antifungal activity was significantly increased by ThPV1, significant increase in the activity of antifungal enzyme of β-1,3-glucanase was observed in the ThPV1-infected strain, and heat treatment reversed the increased antifungal activity of ThPV1-infected strain, the antifungal activity was likely mediated by a hydrolytic enzyme, such as β-1,3-glucanase. ThPV1 modulates the activity of β-1,3-glucanase but not that of chitinase. These results suggest that ThPV1 influences the activity of a specific fungal enzyme, which enhances the mycoparasitism of ThPV1-infected *T. harzianum*. Mycoviruses can exert complex effects on their fungal host, which mediated osmotic stress tolerance (Nerva et al., 2017) and mycotoxin accumulation (Nerva et al., 2018). Thus, whether ThPV1 exerts antagonistic effects on other fungal strains or species should be the subject of further studies.

In addition to the curing experiment including multiple virus-cured and -retained progenies, horizontal viral transmission via hyphal fusion allowed us to verify that the changes in antifungal activities were due to ThPV1. The fact that, compared with the original *T. harzianum* NFCF319 strain, all the viral-transmitted isolates showed similar levels of antifungal activity, such as the growth inhibition of other fungi, indicates that ThPV1 is responsible for the enhanced antifungal activity of the host fungus. Although further work is required, it is important to note the increased β-1,3-glucanase activity is implicated in the hydrolysis of pathogenic fungi during mycoparasitic attack by *T. harzianum* (Qualhato et al., 2013). The antifungal activity was higher in the culture filtrate of ThPV1-infected strains, suggesting that the enhanced β-1,3-glucanase activity influences the potential antifungal activity of ThPV1-infected strains.

To our knowledge, this is the first report of a betapartitivirus in *T. harzianum*. In addition, the mycovirus enhanced the antifungal activity of the host fungus by regulating the activity of a specific antifungal enzyme.

## Author Contributions

D-HK supervised the experiments. JC and H-EY performed the experiments. D-HK, JC, and H-EY prepared the figures and edited the manuscript. D-HK wrote the manuscript.

## Conflict of Interest Statement

The authors declare that the research was conducted in the absence of any commercial or financial relationships that could be construed as a potential conflict of interest.
