# Supplementary material for: Identification of a Novel Partitivirus of Trichoderma harzianum NFCF319 and Evidence for the Related Antifungal Activity
Source: Front Plant Sci. 2018 Nov 20;9:1699. doi: 10.3389/fpls.2018.01699 (PMC6255973; doi:10.3389/fpls.2018.01699)
Supplement: Supplementary file 1 [file Table_1.DOCX]

**Table S1.** Oligonucleotides used in this study.

| Primer name | Sequence (5’ → 3’) |
| --- | --- |
| NFCF319r_F | GGCTTCATCTACCGCACAAT |
| NFCF319r_R | CCAGTCCAGCAGACTGCATA |
| NFCF319c_F | TGCAATCCCTAACGTTTTCC |
| NFCF319c_R | GTCGAGAAAACGGAGACTGG |
| RdRp_F | ACGCCCATCACAGTAAGGAC |
| RdRp_R | CTTGCATACCACCACGAATG |
| CP_F | ATCGCACATGCTCTGTTCTG |
| CP_R | GGACGAGAATGTGCCAATTT |

**Table S2.** Information on the virus used for phylogenetic analysis.

| Reference strain | Abbreviation | GenBank accession no. |
| --- | --- | --- |
| Beet cryptic virus 1 | BCV1 | EU489061 |
| Carrot cryptic virus | CarCV | FJ550604 |
| Cherry chlorotic rusty spot associated partitivirus | CCRSAPV | AJ781401 |
| Chondrostereum purpureum cryptic virus 1 | CpCV1 | AM999771 |
| Dill clover cryptic virus 1 | DCV1 | KF484726 |
| Diuris pendunculata cryptic virus | DpCV | JX156424 |
| Flammulina velutipes browning virus | FvBV | AB465308 |
| Heterobasidion partitivirus 1 | HetPV1 | HQ541323 |
| Heterobasidion partitivirus 3 | HetPV3 | FJ816271 |
| Raphanus sativus cryptic virus 1 | RsCV1 | AY949985.2 |
| Red clover cryptic virus 1 | RCCV1 | KF484724 |
| Rhizoctonia solani dsRNA virus 2 | RHsdRV2 | KF372436 |
| Rosellinia necatrix partitivirus 2 | RnPV2 | AB569997 |
| Sclerotinia sclerotiorum partitivirus S | SsPV-S | GQ280377 |
| Vicia cryptic virus | VCV | AY751737 |
| White clover cryptic virus 1 | WCCV1 | AY705784 |
| Atkinsonella hypoxylon virus | AhV | L39125 |
| Cannabis cryptic virus | CanCV | JN196536 |
| Ceratocystis resinifera virus 1 | CrV1 | AY603052 |
| Crimson clover cryptic virus 2 | CCCV2 | JX971982 |
| Dill cryptic virus 2 | DCV2 | JX971984 |
| Fusarium poae virus 1 | FpV1 | AF047013 |
| Heterobasidion partitivirus 2 | HetPV2 | HM565953 |
| Heterobasidion partitivirus 7 | HetPV7 | JN606091 |
| Heterobasidion partitivirus 8 | HetPV8 | JX625227 |
| Hop trefoil cryptic virus 2 | HTCV2 | JX971980 |
| Pleurotus ostreatus virus 1 | PoV1 | AY533038 |
| Primula malacoides virus 1 | PmV1 | EU195326 |
| Red clover cryptic virus 2 | RCCV2 | JX971978 |
| Rhizoctonia solani virus 717 | RHsV717 | AF133290 |
| Rosellinia necatrix partitivirus 1 | RnPV1 | AB113347 |
| Sclerotinia sclerotiorum partitivirus 1 | SsPV1 | JX297511 |
| White clover cryptic virus 2 | WCCV2 | JX971976 |
| Ustilaginoidea virens partitivirus 1 | UvPV1 | KC503898 |
| Verticillium dahliae partitivirus 1 | VdPV1 | KC422244 |
| Botryotinia fuckeliana partitivirus 1 | BfPV1 | AM491609 |
| Aspergillus fumigatus partitivirus 1 | AfuPV1 | FN376847.3 |
| Discula destructiva virus 2 | DdV2 | AY033436 |
| Discula destructiva virus 1 | DdV1 | AF316992 |
| Ophiostoma partitivirus 1 | OPV1 | AM087202 |
| Gremmeniella abietina RNA virus MS1 | GaRV-MS1 | AY089993 |
| Aspergillus ochraceous virus | AoV | EU118277 |
| Penicillium stoloniferum virus S | PsV-S | AY156521 |
| Fusarium solani virus 1 | FsV1 | D55668 |
| Ustilaginoidea virens partitivirus 2 | UvPV2 | KF361014 |
| Colletotrichum acutatum RNA virus 1 | CaRV1 | KC572132 |
| Penicillium stoloniferum virus F | PsV-F | AY738336 |
| Rose cryptic virus 1 | RoCV1 | EU413666 |
| Fragaria chiloensis cryptic virus | FcCV | DQ093961.2 |
| Raphanus sativus cryptic virus 2 | RsCV2 | DQ218036 |
| Fig cryptic virus | FCV | FR687854 |
| Pepper cryptic virus 1 | PepCV1 | JN117276 |
| Raphanus sativus cryptic virus 3 | RsCV3 | FJ461349 |
| Beet cryptic virus 2 | BCV2 | HM560703 |
| Persimmon cryptic virus | PerCV | HE805113 |
| Pepper cryptic virus 2 | PepCV2 | JN117278 |
| Cryptosporidium parvum virus 1 | CSpV1 | U95995 |
